# Evaluating Neighborhood, Social, and Genetic Influences on Precursors of Alcohol Use Risk Behavior in African American Adolescents

**DOI:** 10.3390/ijerph16173078

**Published:** 2019-08-24

**Authors:** Kimberly S. Compton, Peter Barr, Katherine J. Karriker-Jaffe, Cristina Bares, Fazil Aliev, Brian Mustanski, Danielle Dick, Karen G. Chartier

**Affiliations:** 1 School of Social Work, Virginia Commonwealth University, Richmond, VA 23284-9106, USA; 2 Department of Psychology, Virginia Commonwealth University, Richmond, VA 23284-9106, USA; 3 Alcohol Research Group, Public Health Institute, Emeryville, CA 94608-1010, USA; 4 School of Social Work, University of Michigan, Ann Arbor, MI 48109-1106, USA; 5 Department of Medical Social Sciences, Northwestern University, Chicago, IL 60611-3234, USA; 6 Department of Human and Molecular Genetics, Virginia Commonwealth University, Richmond, VA 23284-9106, USA; 7 Department of Psychiatry, Virginia Commonwealth University, Richmond, VA 23284-9106, USA

**Keywords:** adolescents, externalizing behaviors, family stressors, racial discrimination, neighborhood, genetic risk

## Abstract

*Background*: Using a socioecological framework, we examined neighborhood and social stressors in concert with genetic risk for alcohol dependence in relation to externalizing behaviors, important precursors to alcohol-related problems. *Methods*: We used data from African American adolescents and their caregivers in the Gene, Environment, and Neighborhood Initiative, a subsample of the Mobile Youth and Poverty Study. Participants for the current analyses included 112 adolescents who reported ever having at least one full drink of alcohol. Empirical Bayes scores were used to estimate neighborhood-level violence and transitions. Multivariate models tested main effects and then interactions of family stressors, discrimination, and genetic risk with the neighborhood variables. *Results*: In the main effects model, adolescent externalizing behaviors were positively associated with greater family stressors, more racial discrimination experiences, and genetic liability, while neighborhood variables were nonsignificant. We found three significant interactions. Specifically, the joint effects of neighborhood violence and transitions and between these neighborhood variables and family stressors were significantly associated with externalizing behaviors. *Conclusions*: Our findings suggest genetic liability and complex interactions between neighborhood context and social stressors are important contributors that should be considered in the development of early prevention programs for adolescents who live in economically disadvantaged areas.

## 1. Introduction

African American adults who drink are disproportionately burdened by the consequences of alcohol use [1,2]. They are more likely to binge drink than Whites [3] and are at increased risk of alcohol-attributed social and health problems including relationship violence and heart disease [2]. This is in contrast to African American adolescents who, compared to other racial/ethnic groups, report a delayed onset of drinking, drink less frequently, and consume fewer drinks on occasions when they do drink alcohol [1,4,5]. The study of precursors of problematic alcohol use in African American adolescents, therefore, is important to help identify putative mechanisms associated with higher risk for drinking problems at later developmental stages, which may serve as possible targets for intervention [6]. As such, the aims for the current study are to examine associations between stress-related factors, genetic influences, and externalizing behaviors. Specifically, we examine variables at three ecological levels, including stressors youth experience from the people they interact with and in the neighborhoods where they live, as well as their genetic risk for alcohol dependence, in association with externalizing behaviors in a sample of African American adolescent drinkers.

Externalizing behaviors are a cluster of behavioral, social, and emotional characteristics that include impulsivity, aggression, and substance use [7] and are a key pathway to later heavy alcohol use and alcohol use disorder [8,9], with children typically demonstrating behavior problems earlier than when they establish risky alcohol use. Genetic studies using twin and family designs have consistently demonstrated that alcohol misuse and other externalizing problems share a common genetic etiology; these studies locate alcohol use disorder within a broader externalizing spectrum [10,11]. Externalizing behaviors also are a relevant outcome for African American adolescents because of their later onset of drinking [1,5], and because of the mediating role externalizing behaviors play in the relationship between social stressors and later substance use [12,13]. Conduct disorders, a clinically elevated subset of externalizing behaviors, also are more prevalent among African American youth compared to other behavioral health conditions [14].

Environmental triggers, or stressors, have been associated with both externalizing problems [15] and alcohol use [16]. Socioecological models [17,18] and developmental contextualism [19] emphasize the importance of family, social, and neighborhood factors in youth development. Key stressors such as family instability [15,20], discrimination experiences [12,21], and neighborhood disadvantage [22] each are risk factors for the development of externalizing behaviors and substance use, with effects on the latter being stronger later in the life course [23]. A “double jeopardy” hypothesis posits that experiencing multiple social stressors may be particularly detrimental [24,25], leading individuals to engage in problem behaviors and to use alcohol as a way of coping [13,26]. For example, experiences of unfair treatment and perceived ethnic stigma are associated with alcohol-related problems in African Americans with incomes below the poverty level, but not in their peers with higher incomes [27]. Previous studies also show factors at the neighborhood level can interact with and influence the impact of individual and social predictors on youth delinquency [15] and alcohol use [28] outcomes.

Genetic vulnerabilities directly increase risk for externalizing behaviors [29] and alcohol use [30], and dual hazards models emphasize that these genetic factors interact with stressful environments that can either reduce or exacerbate environmental risks [16,31], especially in predicting externalizing behaviors [32] and alcohol use [33,34]. Other research has shown stressful environments also may ‘turn on’ specific genetic vulnerabilities to substance use behaviors. Twin and family research show environmental influences on externalizing behaviors are stronger among those living in more disadvantaged neighborhoods [35,36], suggesting high-risk environments play a strong role in the expression of the phenotype. Evidence from studies of high residential mobility [37] and increased alcohol outlet density [38] similarly point to the influence of neighborhood context in enhancing genetic effects for adolescent and young adult alcohol use.

In this study, we build on the reviewed literature by examining the intersection of neighborhood, social risk factors (family stressors and racial discrimination) and genetic risk for alcohol dependence in relation to youth externalizing behaviors, emphasizing interactions of multiple neighborhood variables as well as cross-level interactions of neighborhood risk factors with family stressors, racial discrimination experiences, and genetic risk. We hypothesize that stressors at different ecological levels (neighborhood and social) and genetic risk at the individual level are each significantly associated with youth externalizing behaviors. In accordance with double jeopardy and dual hazards models of youth development, we also expect that neighborhood-level stressors will condition the influence of one another, as well as interact with lower-level variables. That is, we hypothesize exposure to multiple risk factors at the neighborhood level and exposure to multiple risk factors across levels (such as high neighborhood and high family stress or high neighborhood and high genetic risk) will be associated with increased externalizing behaviors among youth who have initiated drinking. To test these double jeopardy and dual hazards hypotheses, we focus on neighborhood violence and neighborhood transitions, as well as family stressors, racial discrimination, and genetic liability, as they are important independent risk factors for externalizing behaviors that have not been examined jointly in this unique sample of African American adolescents, which is an understudied population in genetic research [31].

## 2. Materials and Methods

Participants for this study were African American adolescents (ages 13–18) and their primary caregiver in the Gene, Environment, Neighborhood Initiative (GENI). The GENI study examined the effect of housing relocation, between 2004 and 2005, as a part of a federal program on adolescent mental health and risk behaviors [39]. The GENI sample is a subsample of participants in the Mobile Youth and Poverty Study (MYS) [40], who were selected from 13 of the most impoverished neighborhoods in Mobile, AL [41]. When data were collected, 22.4% of the residents of Mobile were below the poverty level. Previous studies have reported details of the GENI data collection methods [39]. Informed consent from caregivers and youth assent were obtained from all participants included in the study. Protocols were approved by the Institutional Review Boards at the University of Alabama, Northwestern University, and Virginia Commonwealth University.

For this study, we used self-reported adolescent survey data on externalizing behaviors, exposure to violence, family stressors, discrimination experiences, and demographic characteristics and caregiver survey data on family income, neighborhood collective efficacy, and neighborhood physical and social problems. These data were collected from families at one time point over the two-year period from 2009 to 2011. Additionally, participant reported addresses were geocoded, allowing neighborhood residence to be assigned to a corresponding 2010 U.S. Census tract [39]. Genetic data came from saliva samples collected from adolescents using Oragene collection kits and under the supervision of a trained interviewer.

The GENI sample included 592 adolescents; however, some participants had missing responses on key analytic variables for the current analysis and were, consequently, excluded. This included: (1) those who did not provide a saliva sample or, if provided, the sample did not pass DNA and genotyping quality control (QC) (*n* = 70); and/or (2) missing responses on survey data included in the analysis (*n* = 30). There was a subset of individuals with incomplete census information (*n* = 177); a comparison showed that those adolescents with and without complete information were similar on all study variables with the exception of age, with those having complete information being older (M = 16.29, SD = 1.30 versus M = 15.13, SD = 1.39; *t* (583) = 9.62, *p* < 0.001).

Additionally, because of this study’s focus on precursors of potential later alcohol problems and the relationship of genetic risk for alcohol dependence with externalizing behaviors, nondrinkers were excluded (*n* = 354) from the final analytic sample. The subsample eligible for our analyses was 112 youth who reported ever having at least one drink of alcohol. We did this for two reasons. First, in preliminary analyses we found that drinkers were older and had higher externalizing scores than nondrinkers (See Table 1), with higher externalizing behaviors indicating an increased risk for later heavy alcohol use and alcohol use disorder. We also did this to maximize associations of alcohol dependence-related genetic effects with externalizing behaviors in our examination of genetic main effects and interactions [42], and given that twin-based studies show genetics have only a small effect on early alcohol use [43,44]. Bares et al. [45] showed in their study using the GENI sample that alcohol-dependence polygenic risk was not associated with alcohol use or conduct problems when nondrinkers were included in the analysis with drinkers. For the current study, participants who reported ever drinking alcohol initiated drinking at a mean age of 14 years (M = 14.20, SD = 2.78) and had a median drinking frequency and quantity, respectively, of 1 or 2 days in the last 12 months and 2 alcoholic drinks on those days.

### 2.1. Genotyping and Quality Control (QC)

The steps performed for genotyping, imputation, and generating ancestry principal components (PCs) are described briefly here and in more detail elsewhere [46]. Genotyping was conducted at the Rutgers University Cell and DNA Repository using the Affymetrix Biobank array (653 k). The array has both a framework for imputation of common genome-wide association study (GWAS) variants (296 k) and functional variants (357 k) that include rare, high-impact exome variants (272 k), indels (18 k), eQTLs (16 k), and miscellaneous markers (51 k). QC checks were standard, similar to those implemented for the Psychiatric Genetics Consortium [47]. Both poorly performing samples and variants were removed. These included samples missing >2% of genotypes, off-target variants found by SNPolisher, single nucleotide polymorphisms (SNPs) missing >5% genotypes, and SNPs missing >2% of genotypes after sample filtering. Imputation was carried out using the remaining 560,138 markers, a combination of SHAPEIT2/IMPUTE2, and the 1000 Genomes Phase 3 (1KGP) reference panel (*n* = 2504; and 81,706,022 variants) [46]. Next, genetic ancestry PCs were generated using the 109,259 semi-independent variants (SNPs associated at r^2^ < 0.10 were retained) common between the 1KGP reference panel and the post-QC GENI sample [46]. A principal component analysis was conducted. Only the 1KGP reference panel was used to determine the SNP weights for each eigenvector. This solution was then projected onto the GENI data to generate 10 ancestry PCs.

### 2.2. Measures

#### 2.2.1. Adolescent Variables

##### Externalizing Behaviors

We used participants’ self-reported externalizing behaviors as the primary outcome for this study. Adolescents completed the 118 item Achenbach Youth Self-Report [7], which assesses a number of problem behaviors within the past 6 months. The externalizing behaviors subscale consisted of 32 items related to aggressive behaviors and rule breaking (α = 0.89) [48]. Summed scores ranged from 0–64. Respondents answered on a 3-point scale from 0 (not at all) to 2 (very or often). Sample items included “I am mean to others”; “I physically attack people”; “I break rules at home, school, or elsewhere”; and “I drink alcohol without my parents’ approval.”

##### Polygenic Scores

Complex genetic traits like externalizing behaviors and alcohol dependence are linked to many genetic markers with small effects [49,50], and polygenic scores (PGS) are an effective tool for aggregating these effects. In this study of precursors of drinking problems, genome-wide PGS were calculated as the sum of alcohol dependence-associated alleles. Because externalizing behaviors are a part of the developmental chain that increases risk for the onset of alcohol-related disorders later in life [8,9], these early risk behaviors are relevant to this study focusing on African American adolescents, a group with a typically delayed onset and low frequency of alcohol use [1,4]. PGS were weighted by summary statistics from a GWAS of alcohol dependence conducted in a sample of 4629 African American adult participants [30]. SNPs common between the current study and the GWAS were, first, grouped around highly significant index SNPs and then pruned out based on linkage disequilibrium (LD) using the ‘clump’ command [51] in Plink [52]; a 500 kb range and LD threshold of r^2^ ≥ 0.25 were applied. Using the remaining semi-independent SNPs, polygenic scores were calculated in PLINK as a linear function of the number of alcohol dependence associated alleles that each adolescent possessed, weighted by the product of the sign of the SNP effect (positive or negative) and the negative logarithm (base 10) of the associated GWAS *p*-value. Nine scores were constructed at *p*-value inclusion thresholds from *p* < 0.50 to *p* < 0.0001. In preliminary analyses, the score using the *p* < 0.01 threshold accounted for the most variance in externalizing behaviors in the current sample, so it was used for all subsequent analyses.

##### Family Stressors

Youth reports of exposure to family transitions in the previous 12 months come from the Stress Index [53]. The life transitions subscale included six items (α = 0.51 in the broader GENI sample) asking adolescents to indicate the frequency (0, never; 1, once; 2, twice; 3, three times or more) with which they were exposed to a variety of family transitions. Items included moving to a new residence, family member getting married, new baby, someone moved out of home, changing schools, and living in a foster home. Summed scores range from 0 to 18 [53]. An earlier GENI study focused on changes associated with being in the housing relocation intervention group [39], while the current study examines a broader set of family transitions.

##### Racial Discrimination Experiences

The Appraisal subscale of the Schedule of Racist Events [54] measured adolescents’ perception of level of stress (i.e., “how stressful was this for you?”) in the previous 12 months related to incidents of racism and being treated unfairly by select groups of people, for example, teachers, other students, doctors, clerks, waiters, neighbors, and police. The subscale included 14 items (α = 0.94) [54]. The response categories ranged from 0 (not at all) to 3 (very), with summed total scores of 0 to 28.

##### Neighborhood Violence and Neighborhood Transitions

The two neighborhood-level variables in this study were based on adolescent self-report data that were aggregated up to the U.S. census tract level. This approach was informed by the work of developmental theorists and sociologists who recognized that individual behavior is strongly influenced by systems and the environment [18], and that characteristics of environments are made up of individual experiences that reflect more than the sum of their parts [55]. As such, individual experiences, aggregated to the neighborhood level, were conceived as a characteristic of the neighborhoods where the adolescents lived [56].

For the first variable, adolescents completed the Exposure to Violence assessment to evaluate exposure to neighborhood violence in the previous 12 months. The scale had nine items, coded 0 = no and 1 = yes, and summed scores ranged from 0 to 9 (α = 0.69) [57,58]. Sample items included whether anyone was robbed or attacked, stabbed/cut, mugged, or sexually assaulted “… in your neighborhood” in the previous 12 months. The second variable, neighborhood transitions, utilized the self-report data for the family stressors variable (as described above), aggregated to the neighborhood level.

We created the neighborhood-level scales using an ecometric approach [59,60]. Methodologically, the ecometric approach uses the responses of individuals in a neighborhood (census tract) to estimate what the true value is for neighborhood violence and neighborhood transitions by modeling the person-level and neighborhood-level variances. We fit a three-level logistic regression with scale items nested within individuals who were nested within neighborhoods, allowing all GENI respondents with any survey data on their neighborhood context to contribute data to the score, regardless of whether they had complete responses on all measures. Scores were calculated from the estimates of posterior means (empirical Bayes or BLUP scores) of the random effects at the neighborhood level. We represented each neighborhood’s deviation from the grand mean across all neighborhoods included in the sample (*N* = 41), in accordance with previous research on neighborhoods and health [61,62,63]. Items were then standardized and coded so that higher scores indicated greater levels of neighborhood violence or more neighborhood transitions.

#### 2.2.2. Caregiver Variables

##### Family Income

Caregivers answered the question, “How much money did you live on during the last year?” by selecting from six response categories: 1 = under $10,000, 2 = $10,000–$19,999, 3 = $20,000–$29,999, 4 = $30,000–$39,999, 5 = $40,000–$49,999, and 6 = $50,000 and above. The modal response was under $10,000 per year, so we used a dichotomous indicator of having an income of $10,000 or more. The family income variable was used in post hoc analyses only.

##### Collective Efficacy

Collective efficacy, the sense that neighbors can work towards achieving a common goal or tackling a common problem, has been shown to protect against adolescent substance use even when individuals have been exposed to violence in the neighborhood [64]. Caregivers’ current perceptions of neighborhood informal social control and social cohesion and trust used a 10-item scale [56,65]. Informal social control items included, “How likely is it that your neighbors would get involved or intervene if children were showing disrespect to an adult?” and “How likely is it that your neighbors would get involved or intervene if a fight broke out in front of your house?”. Response categories were 5 = very likely to 1 = very unlikely. Social cohesion and trust items included, “People around here are willing to help their neighbors” and “People in this neighborhood can be trusted” (5 = strongly agree to 1 = strongly disagree). We computed collective efficacy as the mean of all items on both of these scales. Scores ranged from 1 to 5, with higher scores indicating higher levels of collective efficacy (α = 0.80) [56]. This variable was used in post hoc analyses only.

##### Neighborhood Ecology

Caregivers indicated whether they agreed or disagreed with a series of questions about their current neighborhood and relationships with their neighbors, adapted from the Chicago Youth Development Study [15]. The two subscales are sum scores measuring physical (8 items; range: 0–8; α = 0.82–0.85 in high-poverty samples) and social (6 items; range: 0–6; α = 0.86–0.87 in high-poverty samples) neighborhood problems, with higher scores indicating more problems [66]. Examples of physical problems are too much graffiti, too much vandalism, and too many abandoned apartments or buildings. Examples of social problems are too much drug use, too much alcohol use, and too many people hanging around. These variables were used in post hoc analyses only.

### 2.3. Analyses

We ran all analyses in STATA 14 [67]. We first compared drinkers and nondrinkers on study variables using bivariate *t*-tests and chi-squares. Multivariate models, in drinkers only, used linear regression with robust standard errors to account for clustering in the data. We standardized all independent variables before entering them in the model. Analyses controlled for gender and age. We also controlled for ancestral PCs (associated at *p* < 0.05 with externalizing behaviors in preliminary analyses) to control for spurious effects for the polygenic score that could be explained by within-ancestry group population stratification [68] (the random variation in allele frequencies across populations due to genetic drift). To test our hypotheses based on double jeopardy and dual hazards theories, we first examined a main effects model, then the interaction between the two neighborhood variables, and then cross-level interactions between the neighborhood variables with family stressors, discrimination experiences, and genetic risk. Interactions were entered into the model one at a time to reduce model complexity. Plots of mean predicted values were generated to aid in the interpretation of statistically significant interaction effects. Partial eta squared (η^2^*_p_*) was calculated to measure the effect size of main and interaction effects, indicating the variance explained in the dependent variable after partialling out variance explained by other variables in the model. In post hoc analyses, we applied a median split to scores for each variable included in those significant interaction terms involving neighborhood transitions (i.e., low and high neighborhood transitions, neighborhood violence, and family stressors). We examined these combined effects in relation to caregiver-reported family income using chi-square tests and with mean neighborhood collective efficacy and neighborhood physical and social problems using one-way ANOVAs and pairwise comparisons.

## 3. Results

### 3.1. Main Effects

Externalizing behaviors were associated with measures at two different ecological levels: social stressors and genetic liability (see Table 2). At the neighborhood level, youth externalizing behaviors were not significantly associated with violence or transitions. Among social stressors, externalizing behaviors were positively associated with greater family stressors and there was a trend association with having more racial discrimination experiences. At the individual level, externalizing behaviors were positively associated with genetic liability for alcohol dependence. The effect sizes for social stressors and genetic liability were small to moderately sized, [69] explaining 11% (family stressors), 3.5% (racial discrimination), and 5% (polygenic score) of the variation in externalizing behaviors. Conversely, the effects of the neighborhood-level variables were relatively small (1.5%–1.7%).

### 3.2. Interaction Effects

**Figure 1:**
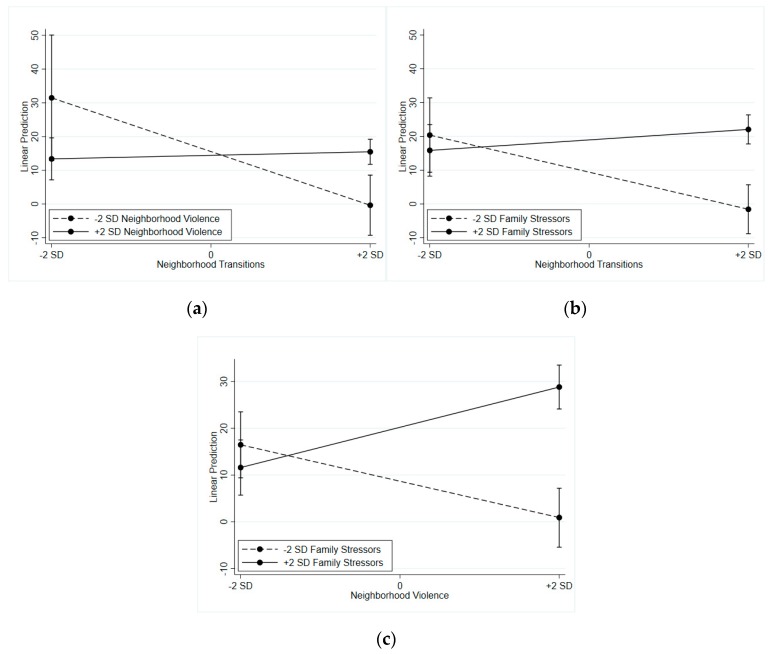
Plots of interaction effects predicting externalizing behavior. (**a**) Interaction between neighborhood transitions and neighborhood violence; (**b**) interaction between neighborhood transitions and family stressors; and (**c**) interaction between neighborhood violence and family stressors.

Three interaction effects were statistically significant, with effect sizes indicating a small to moderate proportion (0.8%, 3.6%, and 6.5%) of variance explained in externalizing behaviors [69]. These were interactions between (1) neighborhood transitions and neighborhood violence (η^2^*_p_* = 0.008), (2) neighborhood transitions and family stressors (η^2^*_p_* = 0.065), and (3) neighborhood violence and family stressors (η^2^*_p_* = 0.036). Figure 1a displays the first interaction effect, showing that neighborhood transitions functioned differently depending on the level of neighborhood violence (B = 2.12, SE = 0.93, and *p* = 0.028). That is, in the context of high neighborhood violence, there was no relationship between neighborhood transitions and adolescent externalizing behaviors. Alternatively, when neighborhood violence was low, increased neighborhood transitions were associated with reduced externalizing behaviors. Figure 1b,c shows the two significant cross-level interaction effects. In Figure 1b, the neighborhood transitions variable also functioned differently depending on the level of family stressors (B = 1.76, SE = 0.75, and *p* = 0.024). A similar pattern emerged as for the first interaction. Under the condition of high family stressors, neighborhood transitions had no effect on externalizing behaviors. There was a protective effect of high neighborhood transitions on externalizing behaviors when family stressors were low. In Figure 1c, the interaction between neighborhood violence and family stressors (B = 2.05, SE = 0.53, and *p* < 0.001) showed a somewhat different pattern. In that, there was a positive relationship between neighborhood violence and externalizing behaviors under the condition of high family stressors, although when family stressors were low, that relationship was negative.

### 3.3. Post Hoc Analyses

**Figure 2:**
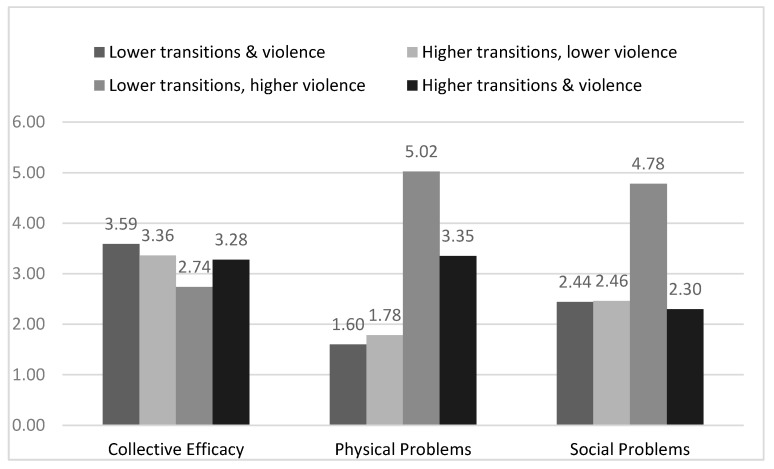
High/low neighborhood transitions and violence: mean neighborhood collective efficacy and physical and social problems.

**Figure 3:**
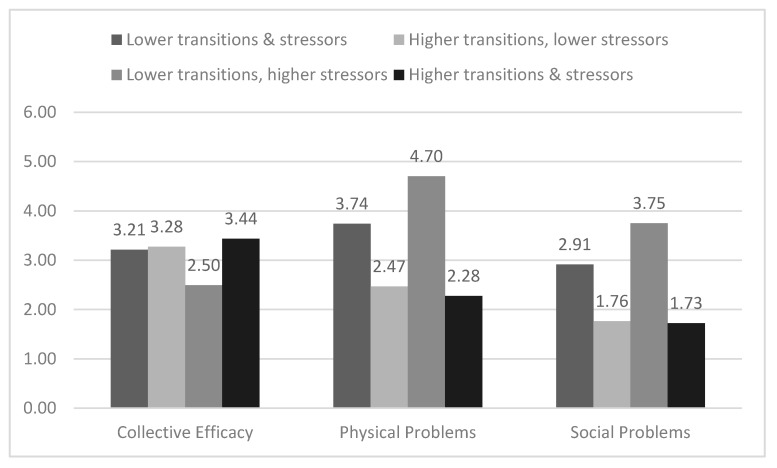
High/low neighborhood transitions and family stressors: mean neighborhood collective efficacy and physical and social problems.

The interaction effect between neighborhood violence and family stressors aligned with our double jeopardy and dual hazards hypothesis; we expected exposure to two risk factors at the neighborhood level or across levels would be associated with higher externalizing behaviors. This was not the case for the two interactions involving neighborhood transitions; therefore, these post hoc analyses sought to address two follow-up questions. The first question was what might explain the weak relationship between neighborhood transitions and adolescent externalizing behaviors for those adolescents who live in higher-violence neighborhoods or who have more family stressors. Figure 2 shows that this could be because lower-transition/higher-violence neighborhoods are associated with a greater level of other risk factors compared to higher-transition/higher-violence neighborhoods. The lower-transition/higher-violence neighborhoods have lower neighborhood collective efficacy than lower-violence neighborhoods (*p* ≤ 0.003) and higher-transition/higher-violence neighborhoods (*p* = 0.032)*,* and those living in lower-transition/higher-violence neighborhoods experience higher levels of neighborhood physical (*p* < 0.001) and social problems (*p* = 0.001) compared to those living in higher-transition/higher-violence neighborhoods. Figure 3 similarly shows that those living in lower-transition neighborhoods with higher family stressors report lower neighborhood collective efficacy (*p* < 0.001) and higher neighborhood physical (*p* < 0.001) and social (*p* = 0.001) problems compared to those living in higher-transition neighborhoods with higher family stressors.

The second follow-up question was what might explain the protective effect of higher neighborhood transitions with externalizing behaviors for adolescents living in lower-violence neighborhoods or with fewer family stressors. The answer to this question is less evident based on the post hoc analyses. For example, living in a higher-transition/lower-violence neighborhood was associated with higher collective efficacy and lower levels of neighborhood physical and social problems (see Figure 2), but these levels were not significantly different from those living in lower-transition/lower-violence neighborhoods (*p* ≥ 0.393). Similarly, in Figure 3, neighborhood collective efficacy was not significantly different between those living in a higher-transition neighborhood with fewer family stressors and those living in a lower-transition neighborhood with fewer family stressors (*p* = 0.807). However, neighborhood physical (*p* = 0.067) and social (*p* = 0.074) problems were marginally lower in higher-transition neighborhoods compared to lower-transition neighborhoods for those with fewer family stressors.

We found similar results when examining family income. Family income was associated with the combined effect of neighborhood transitions and neighborhood violence (*χ*^2^ (3) = 19.92, *p* > 0.001). Participants living in higher-violence neighborhoods, including with lower (27.0%) and higher neighborhood transitions (40.0%), were less likely than participants living in lower-violence neighborhoods (with lower transitions (50.0%) and higher transitions (76.9%)) to have incomes of $10,000 and higher. The relationship between family income and the combined effect of neighborhood transitions and family stressors was not statistically significant (*χ*^2^ (3) = 3.93, *p* = 0.269). For participants with higher family stressors, 35.0% of those living in lower-transition neighborhoods and 55.0% in higher-transition neighborhoods had incomes at or above $10,000 per year. For participants with lower family stressors, 45.7% who lived in lower-transition neighborhoods and 64.7% in higher-transition neighborhoods had incomes at or above $10,000 per year.

## 4. Discussion

In this study of African American adolescents living in high-poverty neighborhoods, we examined main effects and interactions of variables at different ecological levels in association with youth externalizing behaviors. We studied these relationships in individuals who reported drinking alcohol as a way to understand the influence of precursors of alcohol problems that could develop later in the life course. Sample comparisons showed that being older and having higher externalizing behaviors were associated with being a drinker compared to a never drinker of alcohol. Among youth who had used alcohol, externalizing scores were higher when adolescents experienced more family stressors and discrimination experiences, and they had greater genetic liability for alcohol dependence. Our findings are in line with earlier studies showing that social environmental triggers [15,16] and genetic vulnerabilities [29,30] are associated with externalizing disorders and problems with alcohol. Family stressors had the largest influence on externalizing in this study, relative to other variables and neighborhood interaction effects.

Further, although neighborhood violence and neighborhood transitions were not associated with externalizing behaviors in the main effects model, their combined effects and interactions with family stressors were greater than their additive individual effects. The joint effect of living in a higher-violence neighborhood and higher-stressor family was associated with increased externalizing behaviors and supported our double jeopardy hypothesis [24,25]. Alternatively, the joint effects of living in a higher-transition but lower-violence neighborhood or a higher-transition neighborhood but lower-stressor family each were associated with reduced externalizing behaviors, without notable elevations in externalizing behaviors in higher-transition neighborhoods that also were higher on violence or for higher-stressor families. These interaction effects were contrary to our hypothesis that experiencing higher neighborhood transitions in conjunction with either higher neighborhood violence or with higher family stressors would lead to greater externalizing scores.

Post hoc analyses attempted to investigate these unexpected findings by comparing collective efficacy, family income, and physical and social problems across neighborhoods and family stressors. In particular, we wanted to understand why living in a lower-transition but higher-violence neighborhood, or in a lower-transition neighborhood but higher-stressors family, had a similar risk effect for externalizing behaviors as living in a higher-transition, higher-violence neighborhood or in a higher-transition neighborhood and a higher-stressors family. Post hoc tests showed that lower-transition/higher-violence neighborhoods were associated with a greater level of other risk factors (low collective efficacy, low family incomes, and higher levels of physical and social problems) compared to higher-transition/higher-violence neighborhoods. The results were similar when comparing lower-transition neighborhoods/higher-stressor families and higher transition-neighborhoods/higher-stressor families. Therefore, our assumption that lower-transition neighborhoods are more stable and protective [56] was not supported by our post hoc results.

Studies of neighborhood residential mobility may help to explain our findings, in that neighborhood stability is not good for all communities [70,71,72,73]. This is particularly true in disadvantaged neighborhoods, where families have fewer resources to move out. In low-income neighborhoods with low residential mobility, residents experience greater distress, feelings of powerlessness, and being trapped, despite strong social ties among neighbors [70]. This is contrary, in the same study, to the better outcomes (less distress, for example) observed among residents of higher-income neighborhoods with low residential mobility [70]. Additional cross-sectional and longitudinal studies of adults and adolescents further support the notion that higher levels of neighborhood stability and cohesion can result in higher levels of neighborhood violence, in part due to youths’ unstructured time spent with deviant peers [71,72,73], perhaps due to the increased sense of trust among parents in the neighborhood.

Our post hoc tests also were intended to help explain the observed buffering effect of living in neighborhoods with higher transitions but lower violence or with fewer family stressors. However, comparisons of collective efficacy, family income, and physical and social problems did not clarify this finding. The comparisons all were nonsignificant, with the exception of two marginal associations: individuals in higher-transition neighborhoods who also reported lower family stressors had lower neighborhood physical and social problems when compared to the lower-transition/lower family stressors category. The explanation for this protective effect could be related to variables we did not consider, such as underlying mechanisms present in neighborhoods with high violence or high transitions. Other studies find that parental monitoring and family support can mediate effects of living in disadvantaged neighborhoods [15,74], and these deserve further study in relation to neighborhood transitions and neighborhood violence. It also could be that our results are explained by the characteristics of the neighborhoods where study participants reside. As reported previously, families in the GENI sample lived in either very high poverty neighborhoods or less disadvantaged, but still low socioeconomic status, neighborhoods [39]. Prior evaluation of the housing relation program showed that residents in the intervention group had similar family incomes as the control group, and they were unlikely to move from a high-poverty neighborhood to one that had markedly higher socioeconomic status [39].

We also expected that neighborhood-level stressors would interact with genetic risk in association with externalizing behaviors, but this dual hazards hypothesis was not supported. Earlier twin and family studies have shown gene–environment interplay, including interactions of genetic risk with neighborhood disadvantage and other neighborhood exposures in relation to both adolescent externalizing behaviors [35,36] and alcohol use [37,38]. There also is evidence for gene-by-environment relationships from molecular genetic studies for externalizing behaviors [32] and alcohol use [16]. However, similar to the underlying effects noted above, more immediate environmental contexts, including parental monitoring, levels of family support, and contact with deviant peers, may have a stronger interaction with genetic risk in younger samples [49,50,75]. Although we did not find an interaction, genetic risk for alcohol dependence was independently associated with the externalizing behaviors, supporting the putative pathway between externalizing problems and later alcohol problems [8,9]. While individual genetic makeup is not an actionable target for prevention, adjusting for the potential of genetic confounding when assessing neighborhood and social risk factors provides valuable information on possible environmental targets for intervention.

The findings of this study support continued efforts to develop interventions to prevent alcohol use and other externalizing behaviors with African American youth in high-poverty neighborhoods that address not only one ecological level, but multiple levels of influence. Promising interventions aimed at prevention of problem alcohol use may be enhanced by accounting for potential risk factors in African American communities, as well as potential protective factors [6,76]. Context-specific prevention interventions that include many synergistic risk and protective factors are needed. Although some successful models of multilevel interventions exist [77], adapting programs for diverse communities, including for African American youth [78], can be difficult [79], and more work in this area is warranted.

### 4.1. Strengths

The strengths of this study include the richness of the variables available for the GENI study, including genetic and survey data and census information for creating neighborhood-level variables. Further, this study contributes to the literature on environmental risk factors for youth. Given these strengths, we were able to examine within-level neighborhood and cross-level interactions between neighborhood and different ecological levels on an important health risk behavior affecting African American adolescents. Further, we were able to aggregate individual perceptions of stressors at the neighborhood level, which can reduce measurement error [60].

### 4.2. Limitations

Elements of the study limit assertions about causality and generalizability of the findings. The data are cross-sectional, and the sample size is small for those with complete data on study variables. While our sample size provided good statistical power to detect medium and large effects in our main and interaction analyses, it was underpowered to detect small effects [69,80]. Therefore, replication with larger samples would be informative. We reason that it is important to test these relationships in the understudied African American populations to lay the foundation for additional work to be conducted. While this research might also have implications for nondrinkers who exhibit externalizing behaviors, there were significant differences with drinkers even within this small sample. In addition, African American adolescents living in high-poverty neighborhoods in the South may be different from their peers living in other places, including in neighborhoods that are more affluent or in other parts of the U.S. Finally, the neighborhood measures, such as the indicators of neighborhood violence, included relatively low-frequency events, and the variability across neighborhoods may suffer from a high frequency of zeroes (“no violence” responses) [59].

## 5. Conclusions

Consistent with other studies, among African American youth who had used alcohol, externalizing was higher when adolescents experienced more family stressors and discrimination experiences, and they had greater genetic liability for alcohol dependence. The significant interaction between neighborhood violence and family stressors showed that the combined effect of experiencing social stressors at two ecological levels was associated with increased externalizing behaviors. Notably, in this study, there were also significant interactions demonstrating protective effects for neighborhoods experiencing higher levels of transitions for adolescents who also lived in lower-violence neighborhoods or in families with fewer stressors. This protective effect was unexpected, but study findings in this low-income sample point to neighborhoods with lower transitions as being places where there is lower collective efficacy and more social and physical problems.

## Figures and Tables

**Table 1:** Comparison of African American youth reporting drinking and never drinking alcohol.

Variables	Drinkers (*n* = 112)	Non-Drinkers (*n* = 242)
**Adolescent**		
Externalizing Behaviors *	14.80 (9.18)	10.00 (7.66)
Exposure to Violence ^1^	2.59 (1.64)	2.43 (2.14)
Family Stressors ^1^	2.29 (2.15)	2.08 (2.20)
Racial Discrimination	2.19 (3.15)	1.89 (4.19)
Polygenic Risk Score	0.08 (0.01)	0.08 (0.01)
Age *	16.63 (1.22)	16.07 (1.31)
% Female	53.57	54.13
**Caregiver**		
Family Income (%)		
Under $10,000	50.00	52.92
$10,000–$19,999	34.82	32.08
$20,000–$29,999	6.25	9.17
$30,000–$39,999	3.57	3.75
$40,000–$49,999	1.79	1.25
$50,000 and above	3.57	0.83
Neighborhood Collective Efficacy	3.17 (0.95)	3.28 (0.78)
Neighborhood Physical Problems	3.20 (2.48)	3.17 (2.23)
Neighborhood Social Problems	2.46 (2.27)	2.51 (2.03)

Notes: Statistics are mean (standard deviation) or percent; * *p* < 0.001; and ^1^ Variables are measured at the individual level and not yet aggregated to the neighborhood level; these variables at the neighborhood level are standardized.

**Table 2:** Main effects model for neighborhood and social network stressors and genetic risk on externalizing behaviors in African American adolescent drinkers (*n* = 112).

Variables	B (SE)	*p* Value	η^2^*_p_*
Neighborhood violence	1.05 (0.74)	0.167	0.015
Neighborhood transitions	−1.26 (0.81)	0.130	0.017
Family stressors	2.97 (0.87)	0.001	0.111
Racial discrimination	1.91 (1.00)	0.062	0.035
Polygenic risk score	1.73 (0.51)	0.002	0.050

Notes: All independent variables were standardized; models controlled for age, gender, and genetic ancestry; and partial eta squared (η^2^*_p_*) is used to report effect size.
